# Optical Encryption Based on Computer Generated Holograms in Photopolymer

**DOI:** 10.3390/polym13091358

**Published:** 2021-04-21

**Authors:** Taihui Wu, Jianshe Ma, Chengchen Wang, Haibei Wang, Liangcai Cao, Ping Su

**Affiliations:** 1 Department of Precision Instrument, Tsinghua University, Beijing 100084, China; wuth18@mails.tsinghua.edu.cn (T.W.); chengche19@mails.tsinghua.edu.cn (C.W.); 2 Tsinghua Shenzhen International Graduate School, Tsinghua University, Shenzhen 518055, China; ma.jianshe@sz.tsinghua.edu.cn (J.M.); whb20@mails.tsinghua.edu.cn (H.W.)

**Keywords:** optical encryption, photopolymer, computer holographic holograms printing, advanced encryption standard, Gerchberg-Saxton algorithm

## Abstract

An optical encryption method based on computer generated holograms printing of photopolymer is presented. Fraunhofer diffraction is performed based on the Gerchberg-Saxton algorithm, and a hologram of the Advanced Encryption Standard encrypted Quick Response code is generated to record the ciphertext. The holograms of the key and the three-dimensional image are generated by the angular spectrum diffraction algorithm. The experimental results show that large-size encrypted Quick Response (QR) code and miniature keys can be printed in photopolymers, which has good application prospects in optical encryption. This method has the advantages of high-density storage, high speed, large fault tolerance, and anti-peeping.

## 1. Introduction

Information encryption is an important research hotspot in information security today. According to incomplete statistics, cybercrime has caused approximately US $400 billion in economic losses worldwide [1,2]. With the rapid developments of the Internet and artificial intelligence, the field of information security has received more and more attention. Optical encryption is one of the important research directions. Compared with computer encryption and quantum encryption, optical encryption has the advantages of parallelism, high speed, large storage density, and multi-dimensional encryption. In 1995, Refregier et al., proposed a famous image encryption technology based on Dual Random Phase Encoding (DRPE) [3]. Subsequently, various improved DRPE-based optical encryption schemes were proposed [4,5,6,7,8]. However, there are still many important issues that need to be resolved, such as speckle noise, real-time encryption, data storage density, and transmission security [9].

In order to solve these problems, many works have proposed various optical encryption schemes. For example, metasurfaces [10], holographic watermarks [11], diffraction imaging [12], ghost imaging [13], photon counting [14], chaotic character encryption [15], polarized light encoding [16], and compressed sensing [17], etc. Gaurav Verma et al., proposed a biometric key-based asymmetric optical encryption method to solve the problem of key distribution in the optical encryption process [18]. Xinyuan Fang et al., studied the Orbital Angular Momentum Holography for high-security encryption, which has strong selectivity in the spatial frequency domain and can realize ultra-large-capacity holographic information storage [19]. Nikolay N. Evtikhiev and others used a 32,000-frame high-speed Digital Micromirror Device (DMD), combined with a fast digital camera, to create a high-speed optical encryption system [20]. Hosung Jeon et al., proposed a high-resolution binary hologram printing technology, which achieved high-resolution binary holographic printing through DMD [21]. Hafiz Saad Khaliq et al., proposed a multifunctional single-layer element reflective array in order to achieve polarization preservation and spin encryption of the reflected beam [22]. Juan M. Vilardy O. et al., used Collins diffraction transform and nonlinear joint transform correlator to perform optical image encryption, which improved the security of the system against brute force cracking and plaintext attacks [23]. Aimin Yan et al., proposed a multi-image optical encryption method based on angular multiplexing Quick Response (QR) code and spiral phase key [2]. Although the above-mentioned research has made interesting progress, the problems still to face are how to effectively store and transmit complex ciphertext information and avoid network attacks.

In this research, we propose an encryption method based on computer holographic holograms (CGH) in photopolymer. This method can print large-size encrypted QR code, miniature key, and miniature 3D image in photopolymer. First, the ciphertext is generated by using the Advanced Encryption Standard (AES) algorithm, and the ciphertext is encoded by the QR code to form an encrypted QR code. Fraunhofer diffraction was performed by the Gerchberg-Saxton (GS) algorithm to generate a phase-only hologram of the AES encrypted QR code. The phase-only hologram of the key and the three-dimensional (3D) image is generated by the angular spectrum diffraction theory. This research has realized multiple encryption and display through the combination of encrypted QR code, miniature key, and miniature 3D image. The volume holographic characteristics of photopolymers effectively avoids network attacks and improves optical encryption performance.

The rest of the paper is arranged as follows: In Section 2, the design theory and method are introduced, including the GS algorithm and the angular-spectrum layer-oriented method. In Section 3, the computational holographic printing method of large-size encrypted QR code is introduced. In Section 4, the computerized holographic printing method of the miniature image is introduced. Section 5 provides the conclusion.

## 2. Design Theory and Method

### 2.1. Coupled Wave Theory

The volume holographic grating can record and reproduce the wavefront information of 3D object. Kogelnik proposed the classic coupled wave theory in 1969, which can comprehensively analyze the diffraction efficiency of volume holographic gratings [24]. The coupled wave theory is an important theory for analyzing the angle and wavelength selectivity of volume holographic gratings and provides a theoretical basis for the following research on volume holographic gratings formed in photopolymer. In this work, for non-absorption volume holographic gratings, the diffraction efficiency of reflective volume holographic gratings can be calculated according to this theory, and the calculation equations are [24]:

(1) $$\eta_{R}=\frac{sh^{2}\sqrt{\nu^{2}-\xi^{2}}}{sh^{2}\sqrt{\nu^{2}-\xi^{2}}+\left(1-\frac{\xi^{2}}{\nu^{2}}\right)},$$

(2) $$\nu =\frac{\pi d\Delta n}{\lambda \sqrt{cos\theta_{r}cos\theta_{s}}},$$

(3) $$\xi =kd\Delta \theta sin\left(\phi -\theta_{0}\right)/2cos\theta_{s}-k^{2}d\Delta \lambda /8\pi ncos\theta_{s},$$

where $\eta_{R}$ represents the diffraction efficiency of the reflective volume holography, v represents coupling strength, ξ represents the Bragg mismatch, d is the thickness of the recording medium, Δn represents the refractive index modulation, and λ is the wavelength of incident light. $\theta_{r}$ is the angle between the reconstructed light and the direction in the normal to surface of separation air-grating within the dielectric material, and $\theta_{s}$ is the angle between the diffracted light and the direction in the normal to surface of separation air-grating within the dielectric material. $\theta_{0}$ is the Bragg angle, Δθ is the angular offset, and Δλ is the wavelength offset k is the size of the grating vector, ϕ is the tilt angle of the grating vector, and n is the refractive index of the medium.

**Figure 1:**
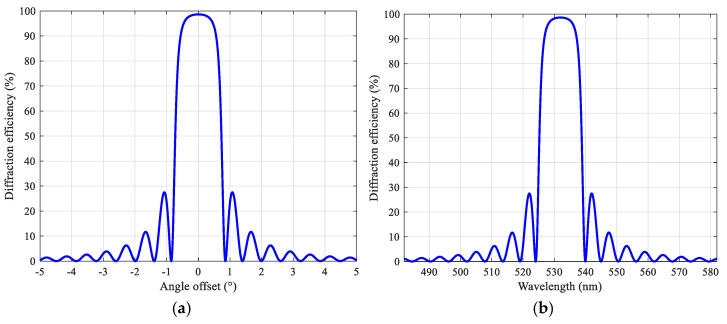
Volume holographic grating: (**a**) angular selectivity; (**b**) wavelength selectivity.

In our configuration, the wavelength is 532 nm, the refractive index of the medium is 1.5, the thickness of the recording medium is 16 μm, the refractive index modulation is 0.03, and the angle between the reference beam and the object beam is 180°. As is shown in Figure 1a, any reference beam that deviates from the recording angle causes a sharp drop in diffraction efficiency, which reflects the angular selectivity of the volume holographic grating. Since volume holographic gratings need to be at a specific angle to observe the strongest diffraction efficiency, this feature plays an important role in encryption.

It can be seen from Figure 1b that when the wavelength of the incident light deviates from the original recording wavelength, the diffraction efficiency will drop sharply to zero. Any reproduced light that deviates from the original recording wavelength will lead to extremely low diffraction efficiency. The peak diffraction efficiency of the main lobe is 98.63%, and the peak diffraction efficiency of the first side lobe is about 27%. This shows that volume holographic gratings have strong wavelength selectivity.

### 2.2. The Gerchberg-Saxton Algorithm

**Figure 2:**
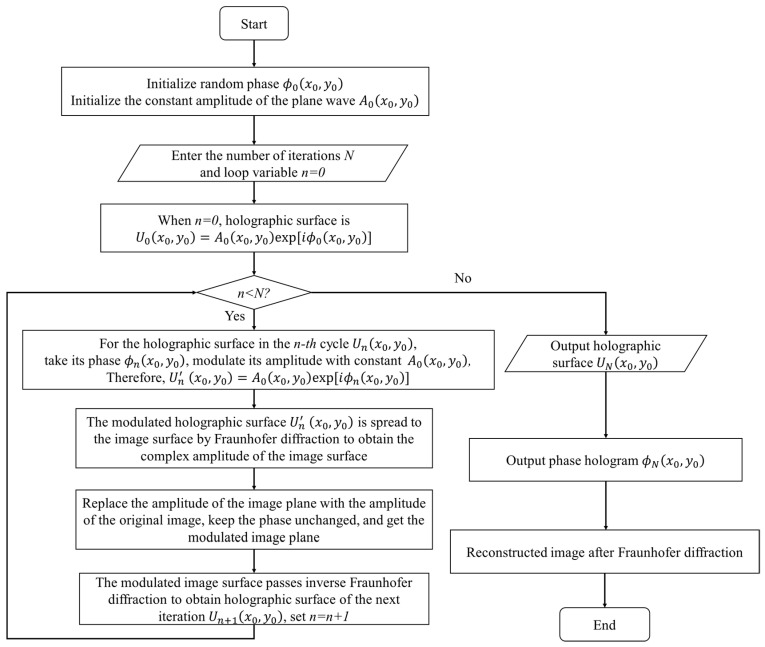
Flow chart of the GS iterative algorithm based on Fraunhofer diffraction.

The GS algorithm was first proposed by Gerchberg and Saxton, which is mainly used for phase recovery [25]. The flow of the GS iterative algorithm based on Fraunhofer diffraction is shown in Figure 2.

The coordinate system (x,y) represents the image surface, and the coordinate system $\left(x_{0},y_{0}\right)$ represents the holographic surface. First, initialize the random phase to $\phi_{0}\left(x_{0},y_{0}\right)$, and the plane wave constant amplitude to $A_{0}\left(x_{0},y_{0}\right)$. When n=0, the complex amplitude of the holographic surface is $U_{0}\left(x_{0},y_{0}\right)=A_{0}\left(x_{0},y_{0}\right)exp\left[j\phi_{0}\left(x_{0},y_{0}\right)\right]$.

For the holographic surface $U_{n}\left(x_{0},y_{0}\right)$ in the *n*-th cycle, take its phase as $\phi_{n}\left(x_{0},y_{0}\right)$. Using the constant $A_{0}\left(x_{0},y_{0}\right)$ to modulate its amplitude, and the modulated complex amplitude is $U_{n}^{\prime }\;\left(x_{0},y_{0}\right)=A_{0}\left(x_{0},y_{0}\right)exp\left[j\phi_{n}\left(x_{0},y_{0}\right)\right]$. In this article, the constant $A_{0}\left(x_{0},y_{0}\right)$ is a matrix of all ones.

The modulated holographic surface $U_{n}^{\prime }\;\left(x_{0},y_{0}\right)$ is spread to the image surface by Fraunhofer diffraction, and the complex amplitude of the image surface can be obtained.

(4) $${\tilde{U}}_{n}\left(x,y\right)=\frac{1}{j\lambda z}exp(jkz+jk\frac{x^{2}+y^{2}}{2z})ℱ\left\{U_{n}^{\prime }\left(x_{0},y_{0}\right)\right\},$$

For the image plane in the *n*-th cycle, take its phase as $\phi_{n}\left(x,y\right)$. Replace the amplitude of the image plane with the amplitude *T* of the original image and keep the phase unchanged to obtain the modulated image plane.

(5) $${\tilde{U}}_{n}^{\prime }\left(x,y\right)=T\cdot exp\left[j\phi_{n}\left(x,y\right)\right],$$

The modulated image surface is inversely diffracted to the object surface through Fraunhofer, and the holographic surface of the next iteration is obtained.

(6) $$U_{n}\left(x_{0},y_{0}\right)=\frac{jexp(-jkz)}{\lambda z}{ℱ}^{-1}\left\{{\tilde{U}}_{n}^{\prime }\left(x,y\right)exp[-\frac{jk}{2z}\left(x^{2}+y^{2}\right)\right\},$$

where ℱ{⋅} represents the Fourier transform, and ${ℱ}^{-1}\left\{\cdot \right\}$ represents the inverse Fourier transform. Repeating the above-mentioned forward diffraction and reverse diffraction process, when *n* = *N*, stop the cycle, and output the hologram.

### 2.3. The Angular-Spectrum Layer-Oriented Method

The angular spectrum diffraction theory is called the plane wave theory of diffraction, which can perform plane wave decomposition [26]. Assuming that the distance between the diffraction surface and the observation surface is *z*, the complex amplitude distributions of the diffraction surface and the observation surface are U(x,y,0) and U(x,y,z), and their corresponding angular spectra are $A_{0}\left(u,v\right)$ and $A_{z}\left(u,v\right)$, respectively. The angular spectrum expressions of the diffraction surface and the observation surface are respectively:

(7) $$A_{0}\left(u,v\right)=\int_{-\infty }^{\infty }\int_{-\infty }^{\infty }U\left(x,y,0\right)exp[-2\pi j\left(ux+vy\right)]dxdy$$

(8) $$A_{z}\left(u,v\right)=\int_{-\infty }^{\infty }\int_{-\infty }^{\infty }U\left(x,y,z\right)exp[-2\pi j\left(ux+vy\right)]dxdy$$

wherein, *u* and *v* represent the spatial frequency of the *x* and *y* axes, respectively. $A_{0}\left(u,v\right)$ and $A_{z}\left(u,v\right)$ are the Fourier transforms of U(x,y,0) and U(x,y,z), respectively. According to the angular spectrum diffraction theory, the relationship between $A_{0}\left(u,v\right)$ and $A_{z}\left(u,v\right)$ is:

(9) $$A_{z}\left(u,v\right)=A_{0}\left(u,v\right)H\left(u,v\right)$$

(10) $$H\left(u,v\right)=exp[jkz\sqrt{1-{\left(\lambda u\right)}^{2}-{\left(\lambda v\right)}^{2}}]$$

wherein, H(u,v) is the transfer function related to the propagation distance *z*. The above equations give the law of angular spectrum propagation, indicating that the angular spectrum on the observation plane is equal to the angular spectrum on the diffraction plane multiplied by a transfer function. After obtaining the angular spectrum of the observation plane, the complex amplitude distribution of the light wave field on the observation plane can be obtained by inverse Fourier transform. In summary, the expression of the complex amplitude distribution in the observation plane is [27].

(11) $$U\left(x,y,z\right)={ℱ}^{-1}\left\{A_{0}\left(u,v\right)\cdot H\left(u,v\right)\right\}={ℱ}^{-1}\left\{ℱ\left\{U\left(x,y,0\right)\right\}\cdot H\left(u,v\right)\right\}$$

The angular-spectrum layer-oriented method is based on the depth map and intensity map of the 3D object to calculate the hologram [27], including three steps of 3D object layering, sub-layer hologram calculation, and hologram superimposition. First, a virtual 3D object is generated by a computer, and its intensity map and depth map are rendered. Then we convert the RGB (Red, Green, Blue) 24-bit image into an 8-bit grayscale image. The layering is performed according to the gray value of the depth map, and the object surface with the same gray value is regarded as the same layer object surface. The distance between each layer of objects is:

(12) $$\varepsilon =\frac{z_{max}-z_{min}}{N}$$

where $z_{max}$ is the maximum depth value, $z_{min}$ is the minimum depth value, *N* is the number of layers, and ε is the spacing between the layers. Therefore, the distance between the object surface of the *i*-th layer and the holographic surface is:

(13) $$z_{i}=z_{0}+i\varepsilon$$

where, $z_{0}$ represents the reference distance between the object surface and the holographic surface (the closest distance). A random phase is added to the amplitude of each layer of the object surface to simulate the scattering effect on the surface of a 3D object. The hologram of the single-layer object surface is calculated through the angular spectrum algorithm, which is $H_{i}={ℱ}^{-1}\left\{ℱ\left(U_{i}\right)\cdot H\right\}$. $H_{i}$ is the hologram of each layer, H is the transfer function of the angular spectrum, and $U_{i}$ is the complex amplitude of the *i*-th layer.

Next, the hologram of each layer is repeatedly calculated, and the complex amplitudes of all sub-layer holograms are superimposed to obtain the complex amplitude distribution of the 3D object hologram. Finally, extract the phase of the hologram, load it into the phase-only spatial light modulator, and irradiate the spatial light modulator (SLM) with coherent light to reconstruct the wavefront of the 3D object.

## 3. CGH Printing of Large Encrypted QR Code

### 3.1. Encrypted QR Code Generation

QR code is a matrix two-dimensional code launched by Japan’s Denso company in 1994 [28]. It is currently the most widely used two-dimensional code encoding method in the world. QR code has the advantages of large data storage capacity, high error tolerance rate, high storage density, low error rate and easy reading, and can encode numbers and English characters. The RS code (Reed-Solomon code) error correction coding mechanism is adopted, and data recovery is realized by adding redundant information to the coded data, which has strong fault tolerance.

Block cipher is one of the important encryption methods of modern cryptography. It has the advantages of fast encryption, standardization, and high security. In block ciphers, the most classic are the AES [29,30]. The AES algorithm has the advantages of security, high efficiency, high flexibility, and strong realizability.

**Figure 3:**
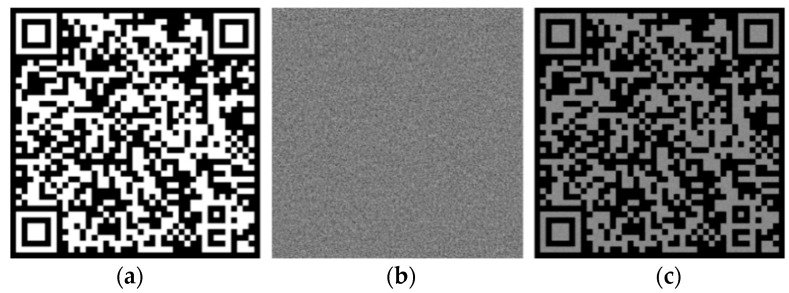
Computational holographic simulation results of encrypted QR code: (**a**) Original image, (**b**) phase-only CGH, (**c**) Numerical reconstruction.

In order to record the ciphertext information in the photopolymer, the ciphertext is encoded by a QR code. The coding system for the QR Code has the error tolerance rate of 30%. The cipher text is generated by the AES algorithm, the plain text is “Product No.: 20210115”, and the key is “Tsinghua19112021”. In the parameter setting of the AES algorithm, the cipher block chaining (CBC) mode is adopted, the padding mode is zero padding, the data block is 128 bits, the encoding method of the output data is mode of Base 64, and the character set is UTF-8. The ciphertext content obtained through AES algorithm encryption is: “4jWsFlO5pipRanYaDFIF7KsOH+5uiY7uwyK4YzzFP3U=”, a total of 44 bytes. The generated QR code is shown in Figure 3a.

We use the GS algorithm to simulate and calculate the phase-only hologram of the QR code key. In the simulation parameter setting, the wavelength is 532 nm, the resolution of CGH is 1080 × 1080, the reconstruction distance is 300 mm, and the pixel size is 8 μm. Through simulation, the phase-only hologram of the encrypted QR code can be calculated, as shown in Figure 3b. It can be seen from Figure 3c that the GS algorithm can be used to reconstruct the QR code key with high quality. Scanning the original image and the numerical reconstruction image through the mobile phone, the scanning results are consistent, indicating that the accurate reconstruction image can be obtained through the GS algorithm.

**Figure 4:**
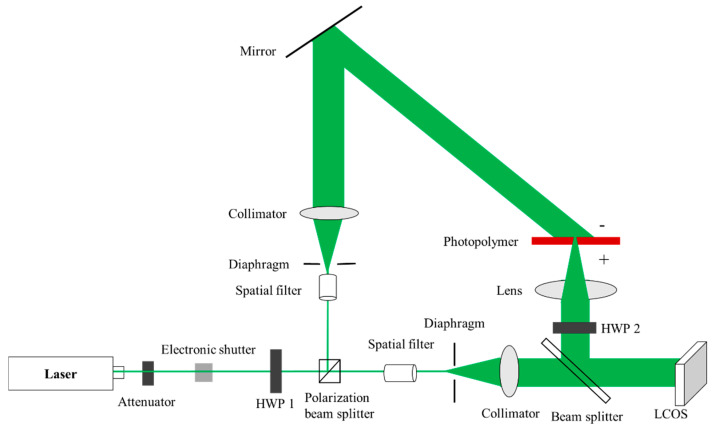
Optical setup for off-axis reflective CGH printing.

The experimental optical setup is shown in Figure 4, and an off-axis reflective CGH printing optical setup is built. The model of the laser is MSL-F-532 (from Changchun New Industries Optoelectronics Technology Co., Ltd., Changchun, China). The laser is a single longitudinal mode laser with a wavelength of 532 nm, an output power of 1 W, and a coherence length greater than 50 m. The emitted laser light passes through the attenuator film, the electronic shutter, and the half-wave plate (HWP) in sequence, and is divided into two polarized light beams. The spatial light modulator used in this study belongs to the type of Liquid Crystal on Silicon (LCOS). Since the phase-type LCOS (from HOLOEYE Photonics AG, Berlin, Germany) can only modulate polarized light, which is used to illuminate the LCOS. The transmitted light beam is P light, which is expanded by a spatial filter (from Daheng optics, Beijing, China), and then becomes parallel light after passing through an aperture and collimation. After the object beam reflected by the LCOS is transformed by a lens (from Hengyang Optics, Guangzhou, China), the background light is focused into a spot. The transform of the object beam through the lens allows the Fraunhofer diffraction pattern to be observed at a close distance. The wavefront modulated beam illuminates the “+” surface of the photopolymer; the reflected light beam is S light, which is irradiated on the “−” surface of the photopolymer after beam expansion, collimation, and reflection. Subsequently, the photopolymer material is moved to the position where the reconstructed image is in focus. In order to improve the recording efficiency, the object beam and the reference beam need to be S-polarized light. Therefore, the HWP 2 (from Zolix Instruments Co., Ltd., Beijing, China) is adjusted to make the object beam reflected by the LCOS to S-polarization.

In this research, the spatial light modulator is LCOS, the model is HOLOEYE PLUTO-2. The pixel of this system is 1920 × 1080, the gray level is 256, the panel size is 15.36 mm × 8.64 mm, the pixel size is 8.0 μm. In recent years, photopolymers have attracted more and more attention in holographic applications due to their good characteristics, such as high diffraction efficiency, large information storage density, high resolution, and low requirements for physical or chemical processing [31,32]. For the selection of photopolymers, the CRT20 holographic film from Litiholo (Newport News, VA, USA) is used. CRT20 holographic film is a photopolymer composite material and is attached to a glass substrate with a thickness of approximately 1.8 mm. The holographic film is composed of two layers, the upper layer is a triacetate cellulose film (TAC) protective layer with a thickness of approximately 50 μm; the lower layer is a photopolymer with a thickness of approximately 16 μm.

Next, we adjust the beam splitting ratio through the half-wave plate HWP 1 (from Zolix Instruments Co., Ltd., Beijing, China), so that the optical power density of the two beams is 3 mW/cm^2^. When performing interference recording, the electronic shutter is opened, the angle between the reference beam and the object beam is 45°, the exposure time is 1 min, and the wavefront information modulated by LCOS is recorded into the photopolymer. After recording, the polymer is placed in darkness for 4 min, then a mercury lamp is applied for 2 min.

**Figure 5:**
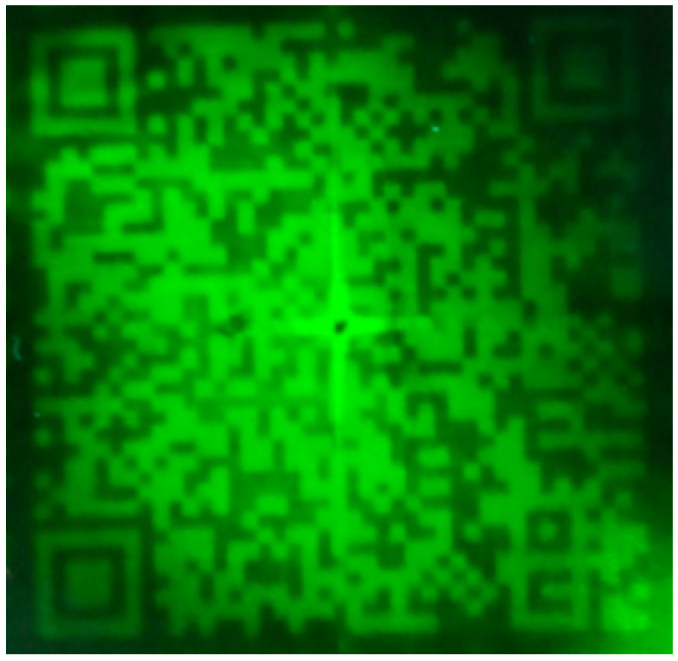
Experimental results of encrypted QR code photopolymer recording.

In Figure 5, the size of the QR code in the photopolymer is 14 mm × 14 mm, and its size is determined by the focal length of the lens. The larger the focal length, the larger the size of the two-dimensional image recorded by the photopolymer. Figure 5 shows the encrypted QR code printed by computer holography, which can be obtained by taking a photo with a mobile phone or camera. Due to the angular selectivity of the volume holographic grating, the encrypted QR code needs to be observed at a specific angle, that is, when the angle between the reproduce beam and the photopolymer surface is 45°. When the angle of the reproduce beam deviates, the diffraction efficiency will drop sharply, and the reconstructed image cannot be observed. Under the irradiation of natural light, the encrypted QR code recorded by the photopolymer is scanned by the mobile phone, and the scanning result is exactly the same as the ciphertext in the original QR code. This shows that the use of photopolymers can accurately record long-length cipher text information, and the cipher text information can be easily found by directly scanning the QR code with a mobile phone. We can use natural light to reproduce 3D objects, because there is a monochromatic component of 532 nm in natural light. Moreover, due to the wavelength selectivity of volume holographic gratings, any waveband deviating from 532 nm can cause a sharp drop in diffraction efficiency and play a filtering role. Due to the Fraunhofer diffraction based on the GS algorithm, the optically reconstructed QR code key has stronger brightness, and a large-size QR code can be obtained, which is conducive to directly scanning the QR code through the mobile phone to obtain the cipher text information.

### 3.2. Reconstruction Quality Evaluation

In this section, we will evaluate the reconstruction quality of the GS algorithm. The root mean square error (RSME) is used to evaluate the reconstruction quality of the image. The smaller the RSME, the better the quality of the reconstructed image. The calculation equation of RSME is

(14) $$RSME=\sqrt{\frac{1}{M\cdot N}\sum \sum {\left(I_{ij}-\overset{\wedge }{I_{ij}}\right)}^{2}}$$

The image size is M⋅N, $I_{ij}$ represents the amplitude of a single pixel in the reconstructed image, and $\overset{\wedge }{I_{ij}}$ represents the amplitude of a single pixel in the original image.

The Peak Signal to Noise Ratio (PSNR) is used to evaluate the optical reconstruction quality of the image, in decibels (dB). The larger the PSNR, the smaller the distortion and the better the quality of the reconstructed image. When the PSNR is greater than 40 dB, the image quality is very good, that is, very close to the original image. The calculation equation of PSNR is

(15) $$PSNR=10\times \log_{10}{\left(\frac{2^{n}-1}{RMSE}\right)}^{2}$$

**Figure 6:**
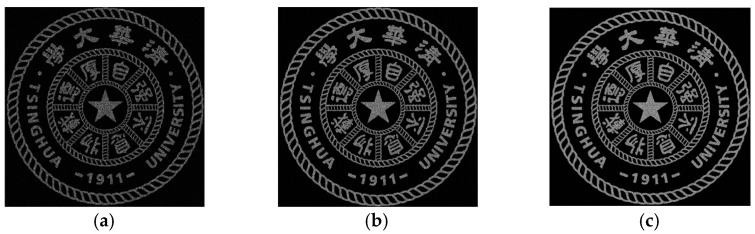
Numerical simulation reconstruction image: (**a**) 1 iteration, (**b**) 10 iterations, (**c**) 100 iterations.

The school emblem of Tsinghua University is used as the original image for hologram calculation. The wavelength is 532 nm, the resolution of CGH is 1080 × 1080, the reconstruction distance is 300 mm, and the pixel size is 8 μm. Figure 6 shows the reconstructed image output from iteration 1, 10, and 100. It can be seen that as the number of iterations increases, the reconstructed image gradually becomes clearer.

**Figure 7:**
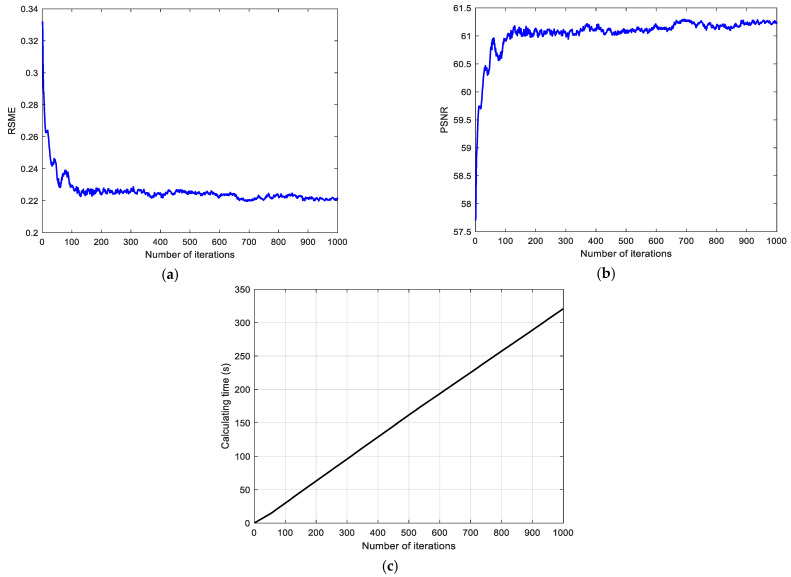
Evaluation of reconstruction quality of GS algorithm: (**a**) RSME, (**b**) PSNR, (**c**) Calculating time.

The RSME and PSNR of the reconstructed image under different iteration numbers are presented. It can be seen that both RSME and PSNR eventually tend to converge within 1000 iterations. In Figure 7a, as the number of iterations increases, RSME decreases rapidly in the first 100 iterations. When the number of iterations is greater than 100, the rate of decline of RSME decreases, and RSME tends to converge to 0.22. In Figure 7b, as the number of iterations increases, the PSNR increases rapidly in the first 100 iterations. When the number of iterations is greater than 100, the growth rate of PSNR decreases, and PSNR tends to converge to about 61. In order to take into account the efficiency of the algorithm, the number of iterations in this article is 100. At this time, the RSME is about 0.22, and the PSNR is about 61. The image reconstruction quality is high and the image distortion is small. In Figure 7c, the number of iterations is proportional to the calculation time. The time consumed for 100 iterations is about 32 s. At this time, both efficiency and reconstruction quality can be taken into account.

## 4. CGH Printing of Miniature Keys

### 4.1. Miniature Key Generation

**Figure 8:**
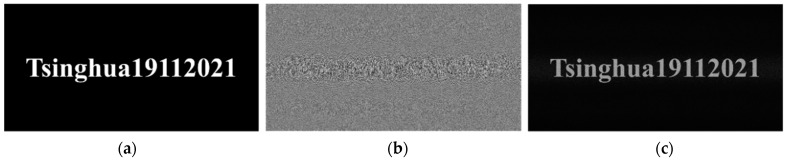
Computerized holographic simulation of the key: (**a**) original image, (**b**) phase-only hologram, (**c**) numerical simulation reconstruction image.

As shown in Figure 8a, for the key “Tsinghua19112021” of the AES algorithm in Section 3.1, we can calculate its hologram through angular spectrum diffraction. In the simulation parameter setting, the wavelength is 532 nm, the resolution of CGH is 1920 × 1080, the reconstruction distance is 220 mm, and the pixel size is 8 μm. Through simulation, the phase-only hologram of the key can be calculated, as shown in Figure 8b. It can be seen from Figure 8c that the GS algorithm can be used to reconstruct the key of the AES algorithm with high quality, and the accurate reconstruction image can be obtained through the GS algorithm.

Using the off-axis reflective computational holographic printing optical path shown in Figure 4, the reconstructed image of the hologram is focused on the surface of the photopolymer, and the recording of the miniature key is finally realized. The only difference is that in this experiment, the lens in Figure 4 focuses the wavefront information modulated by the LCOS at one point to achieve a miniature recording. During the experiment, the laser wavelength was 532 nm, the exposure intensity of the reference beam and the object beam was 3 mW/cm^2^, the exposure time was 1 min, the polymer is placed in darkness for 4 min, and the mercury lamp curing time was 2 min.

**Figure 9:**
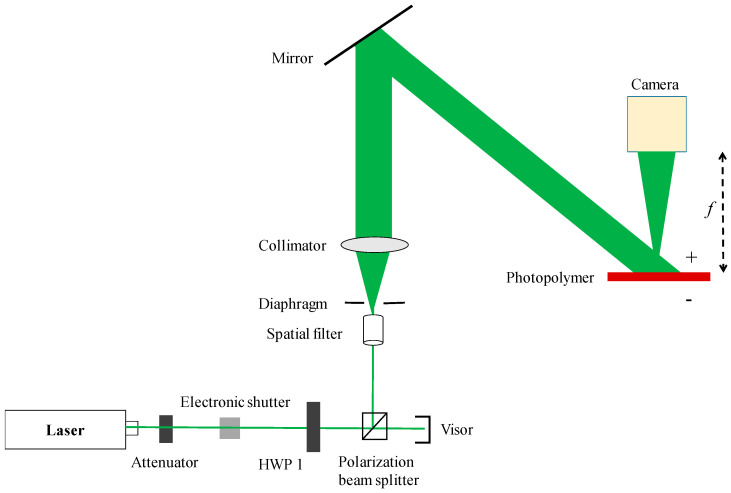
The shooting optical setup of the miniature image.

The optical setup shown in Figure 9 is used to take the micro-image recorded by the photopolymer. The front and back sides of the photopolymer are swapped, and the reference beam remains unchanged. At this time, the photopolymer is irradiated with the reference beam, and the real image can be observed at the symmetrical position of the reconstructed image. The real image can be taken with a camera (Canon 600D).

**Figure 10:**
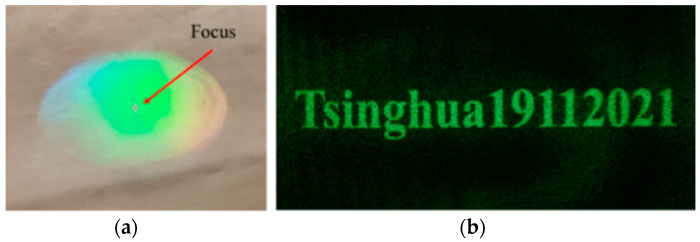
The printed result of the computerized hologram of the miniature key: (**a**) the actual photopolymer, (**b**) a miniature key image taken with a camera under the illumination of the reference beam.

As shown in Figure 10, through angular spectrum diffraction and focusing lens, the miniature key can be recorded in the photopolymer, the reconstructed image is clear, and the stored key is the same as the original key information. We used Charge-coupled Device (CCD) to shoot the focused spot, and the model is MV-EM510M (from Vision Digital Image Technology Co., Ltd., Xi’an, China). After experimental measurements, the spot diameter is 660 CCD pixels and the size of each pixel is 3.45 μm, so the spot diameter is 2.28 mm and the area is 4.08 mm^2^.

### 4.2. Miniature 3D Image Generation

In order to print miniature 3D images in photopolymers, a virtual three-dimensional model is generated by a computer, and a depth map and an intensity map are obtained through rendering. The length of the train model is 30 mm, the width is 5 mm, the height is 10 mm, and the depth is 20 mm. The distance between the train and the hologram is 150–170 mm. In order to facilitate the observation of experimental phenomena, this paper divides the 3D train model into three layers according to the gray value range of the depth map and adds a random phase to each layer.

**Figure 11:**
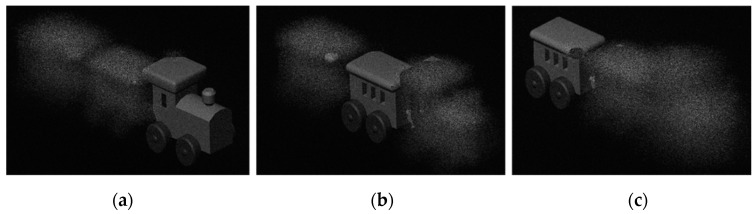
Numerical reconstruction of 3D train model: (**a**) z = 150 mm, (**b**) 160 mm, (**c**) 170 mm.

In the simulation parameter setting, the wavelength is 532 nm, the resolution of CGH is 1920 × 1080, the reconstruction distance is 150 mm, and the pixel size is 8 μm. Through the simulation of the layered angular spectrum algorithm, the phase-only hologram of the 3D image can be calculated. The train model reconstructed by numerical simulation is shown in Figure 11, which represent the reconstructed images when the depth of field is 150 mm, 160 mm, and 170 mm, respectively.

Using the optical path shown in Figure 4, the computational hologram of the 3D object is loaded in the LCOS. During the experiment, the laser wavelength was 532 nm, the exposure intensity of the reference beam and the object beam was 3 mW/cm^2^, the exposure time was 1 min, the polymer is placed in darkness for 4 min, and the mercury lamp curing time was 2 min.

**Figure 12:**
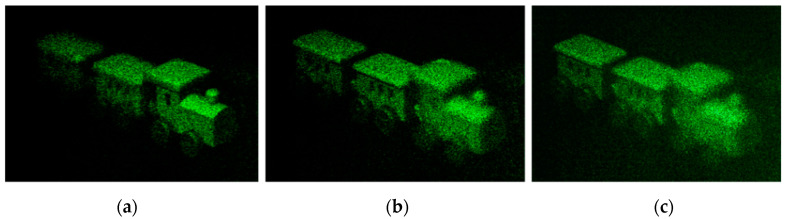
Photopolymer reconstruction of the train: (**a**) z = 200 mm, (**b**) 210 mm, (**c**) 220 mm.

After the recording is completed, the optical setup shown in Figure 9 is used to take a photo of microscopic 3D image recorded in the photopolymer. The reconstruction result is shown in Figure 12. Since the focal length of the focusing lens used is 200 mm, the distance from the camera to the photopolymer plane is determined by the focal length. It can be seen that when the camera is 200 mm, 210 mm, and 220 mm away from the photopolymer surface, the reconstructed image is consistent with the reconstructed image of the numerical simulation, and the clear locomotive, centre and rear of the vehicle can be observed at different depths of field. The experimental results show that the reconstruction of the miniature 3D model can be achieved through the photopolymer.

**Figure 13:**
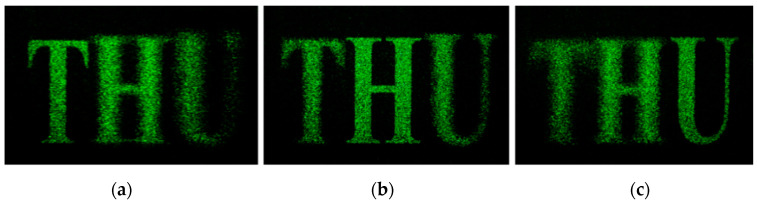
Photopolymer reconstruction “THU”: (**a**) z = 200 mm, (**b**) 210 mm, (**c**) 220 mm.

Figure 13 shows the results of the photopolymer reconstruction of the character string “THU”. The clearest letters ‘T’, ‘H’, and ‘U’ can be captured at different reconstruction distances of z = 200 mm, 210 mm, and 220 mm. The experimental results show that photopolymers can realize the reconstruction of miniature 3D image, and different images can be observed under different depths of field. It can be seen from Figure 12 and Figure 13 that through the encryption of the miniature 3D image, the image with obvious depth information can be recorded into the photopolymer. When irradiated with reproduced beam, when the camera is focused on a certain layer, the other layers become blurred. In this way, the key can be stored in segments, or different keys can be stored in different layers of the material, thereby increasing the information storage density. This method is conducive to the realization of multiple key storage, for example, in asymmetric encryption scenarios where public and private keys need to be used separately. Each layer of keys needs to be recognized under a specific reconstruction distance, and the distance deviation can cause the reconstruction image to be blurred. Each key corresponds to a specific reconstruction distance, and the optical encryption performance is further improved through the combination of the key and the depth of field.

In addition, due to the angular selectivity of the volume holographic grating, the miniature key and the 3D image need to be observed at a specific angle, that is, when the angle between the reproduced light and the photopolymer surface is 45°. When the angle of the reproduced light deviates, the diffraction efficiency will drop sharply, and the reconstructed image cannot be observed. Because it needs to be illuminated at a specific angle to reconstruct the image, the privacy of the key is improved.

### 4.3. Reconstruction Quality Evaluation

In this section, we will evaluate the reconstruction quality of the angular-spectrum layer-oriented method. The reconstruction quality of the algorithm is evaluated according to the PSNR and RSME defined in Section 3.2. We perform numerical reconstruction and calculate the error between the reconstructed image of each layer of the object surface and the original amplitude. The average RSME and average PSNR are used to evaluate the quality of the reconstructed image. The virtual 3D model is used as the original image for hologram calculation. The wavelength is 532 nm, the resolution of CGH is 1080 × 1080, and the pixel size is 8 μm.

**Figure 14:**
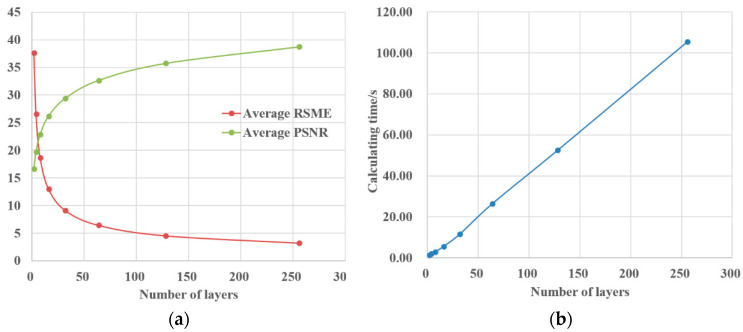
Reconstruction evaluation of the angular-spectrum layer-oriented method: (**a**) The relationship between the number of layers, average RSME and average PSNR, (**b**) The relationship between the number of layers and calculation time.

It can be seen from Figure 14 that as the number of layers increases, the average RSME of the 3D object gradually decreases, and the average PSNR gradually increases. This shows that the larger the number of layers, the higher the quality of the reconstructed image and the smaller the distortion rate, but at the same time the calculation takes longer. Therefore, a compromise can be made between calculation time and reconstruction quality. In this research the number of layers is selected to be 64. At this time, the time to calculate the 3D object hologram is 26.43 s, the average RSME is 6.41, and the average PSNR is 32.66. The simulation results show that the calculation time of the angular-spectrum layer-oriented algorithm is shorter, the image reconstruction quality is higher, and the distortion rate is lower.

## 5. Conclusions

In this research, we propose a QR code encryption and display method based on photopolymer computer holographic printing. This method can record large-size encrypted QR codes, miniature keys, and miniature 3D images in the photopolymer. The ciphertext is generated using the AES algorithm, and the ciphertext is encoded by the QR code to form an encrypted QR code. Fraunhofer diffraction was performed by the GS algorithm to generate a phase-only hologram of the AES encrypted QR code, then through angular spectrum diffraction to generate the key and the phase-only hologram of the 3D image. For the GS algorithm based on Fraunhofer diffraction, RSME is 0.22 and PSNR is about 61. For the angular-spectrum layer-oriented method, when the number of layers is 64, the average RSME is 6.41, and the average PSNR is 32.66. Both of the two hologram generation algorithms have high image reconstruction quality and lower distortion rate, meeting the requirements of optical encryption. Through computerized holographic printing, encrypted QR codes of large size can be obtained, and the cipher text can be quickly scanned through a mobile phone. The spot diameter of the recorded miniature key and miniature 3D image is about 2.28 mm, which effectively realizes the miniature encryption and improves the information storage density of the key. The miniature key and miniature 3D image have good privacy and can play a good role in preventing peeping. In this study, the combination of encrypted QR code, miniature key and miniature 3D image has enhanced the ability to resist attacks and brute force cracking and improved the performance of optical encryption.

## Data Availability

The data presented in this study are available on request from the corresponding author.
